# Bilateral symmetric dry gangrene of the feet in a newborn

**DOI:** 10.11604/pamj.2023.46.26.34933

**Published:** 2023-09-18

**Authors:** Renu Bharat Rathi, Arun Naphe Khatri

**Affiliations:** 1 Department of Kaumarbhritya, Mahatma Gandhi Ayurved College Hospital and Research Centre, Datta Meghe Institute of Higher Education and Research, Sawangi, Wardha, Maharashtra, India

**Keywords:** Feet gangrene, neonate, bilateral, symmetrical, icterus

## Image in medicine

Gangrene is extremely rare in neonates caused by the sudden loss of arterial blood supply to usually distal tissue like limbs/feet and toes. There are three types of gangrene-dry, wet and gas. Wet gangrene when the tissue is infected by saprogenic microorganisms and occurs in naturally moist tissue and organs such as the mouth, bowel, lungs, cervix and vulva. Gas gangrene is a bacterial infection that produces tissue gas in gangrene. This deadly form of gangrene is usually caused by Clostridium perfringens bacteria. In this case, the newborn was full-term normal delivery born with a 2.6kg birth weight. The mother has put socks on both feet of the neonate tied with tight thread to avoid cold exposure. The next day of the discharge on day 3, the parents brought the newborn for icterus and during the examination, the physician noticed the bilateral gangrene present with a line of demarcation at the junction between healthy and gangrenous parts. Parents were counselled for the emergency operation to amputate the feet.

**Figure 1:**
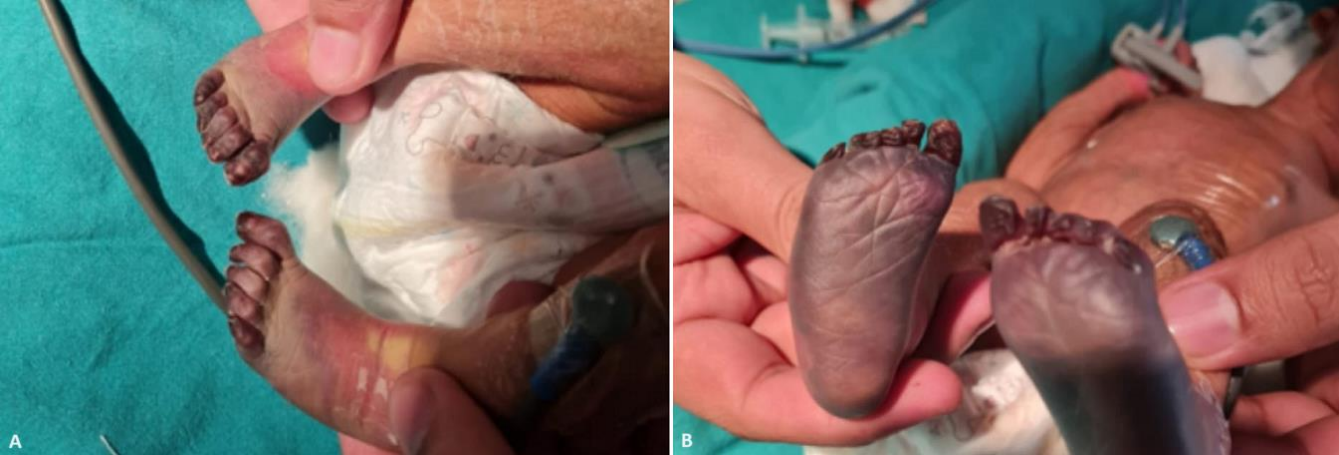
A,B) blackish discolouration and a line of demarcation between the healthy and gangrenous parts of the feet are seen

